# 2942. Successful Screening, Linkage to Care, and Treatment of Hepatitis C in a Tiny Shelter Encampment on Veterans Affairs Grounds

**DOI:** 10.1093/ofid/ofad500.181

**Published:** 2023-11-27

**Authors:** Cassandra C Lautredou, Kimberly A Lynch, Katherine Stricker, Peter Capone-Newton, Matthew McCoy, Jenna Kawamoto, Arpan Patel, Michele Seckington, Debika Bhattacharya

**Affiliations:** UCLA Health, Santa Monica, CA; VA Greater Los Angeles Healthcare System, Los Angeles, California; UCLA Health, Santa Monica, CA; Veterans Affairs Greater Los Angeles Healthcare System, Los Angeles, California; US Department of Veterans Affairs, Los Angeles, California; VA Greater Los Angeles Healthcare System, Los Angeles, California; University of California, Los Angeles and VA Greater Los Angeles Healthcare System, Los Angeles, California; Veterans Affairs, Long Beach, California; University of California, Los Angeles, Greater Los Angeles Veteran Affairs, Los Angeles, CA

## Abstract

**Background:**

Persons experiencing homelessness have a high prevalence of hepatitis C virus (HCV) infection and multiple barriers to diagnosis and treatment. We implemented and evaluated a universal HCV screening and HCV care coordination and treatment program for Veterans living at a tiny shelter encampment on the West Los Angeles Veterans Affairs campus.

Hepatitis C Cascade of Care

**Figure:**
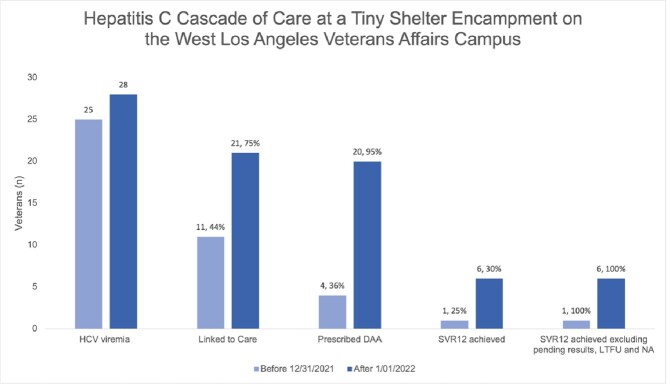

All patients who completed treatment and obtained end of treatment lab work achieved SVR12, excluding Veterans who were lost to follow up (LTFU) and/or non-adherent (NA) (n=5), on treatment (n=4), or SVR12 pending (n=5).

**Methods:**

We implemented a low-barrier to entry HCV treatment program for Veterans experiencing homelessness (VEH) that spanned April 2020-Dec 2021 (pre-intervention) and Jan 2022-April 2023 (post-intervention). The intervention involved a novel e-consult direct-to-treatment program facilitated by pharmacists and referral to ID/GI clinics (if ineligible for e-consult) and extensive engagement by encampment providers, including provision of direct-acting antivirals (DAAs) in weekly installments. Prior to the intervention, management was achieved solely through ID/GI clinic referrals. We evaluated HCV prevalence, along with linkage to care, treatment initiation, and sustained virologic response (SVR) of this novel program pre-and post-intervention. Data, including demographics, medical history healthcare visits, and other laboratory values were abstracted from the electronic medical record. Descriptive statistics were used to characterize prevalence of HCV and other covariates.

**Results:**

Of the 704 Veterans who sheltered at the encampment at some point from April 2020-April 2023, 587 underwent HCV antibody screening (83%). Of those, 20% (120/587) were HCV Ab positive and 44% (53/120) had HCV viremia. Median age of those who were viremic was 64 years, 100% were male, 32% (17/53) were Black, 9% (5/53) Hispanic, and 58% (31/53) White. Pre- and post-intervention, 25 and 28 Veterans with HCV viremia were identified, 11 and 21 Veterans were linked to care (44% and 75%), 4 and 20 initiated DAAs (36% and 95%), and 1 and 6 achieved SVR (25% and 30%). Nine patients are currently on treatment (n=4) or pending SVR12 (n=5).

**Conclusion:**

After an intervention involving e-consult and encampment provider engagement in VEH, 75% of Veterans with HCV viremia were linked to care, compared to 44% of Veterans prior to the intervention. Our program demonstrates the feasibility of encampment-based HCV screening, linkage to care and treatment.

**Disclosures:**

**Debika Bhattacharya, MD, MSc**, Gilead Sciences: Grant/Research Support

